# Need for a single standardized licensing & residency-entrance level exam policy in the medical education system of Pakistan

**DOI:** 10.12669/pjms.38.5.6464

**Published:** 2022

**Authors:** Syed Muhammad Hammad Ali, Hafiz Muhammad Umar Masood, Asim Malik

**Affiliations:** 1 Dr. Syed Muhammad Hammad Ali Senior Intern, Department of Surgery, FMH College of Medicine & Dentistry, Lahore, Pakistan; 2 Dr. Hafiz Muhammad Umar Masood Intern, Department of Surgery, FMH College of Medicine & Dentistry, Lahore, Pakistan; 3 Prof. Dr. Asim Malik Chair, Department of Surgery, FMH College of Medicine & Dentistry, Lahore, Pakistan

The undergraduate medical education system of Pakistan is struggling with the deadline set by the World Federation of Medical Education (WFME) which requires the system to get accreditation with the body by 2024.^1^ Consequently, the highest accreditation and regulation body of medical education; the Pakistan Medical Commission (PMC), is undertaking reforms in this regard. A new form of exit exam for medical and dental graduates is being devised and conducted across Pakistan with an aim to place a quality-check on the teaching standards of medical schools and universities across the country.

Pakistan is the third largest provider of clinicians to the United States of America from around the world.^2^,^3^ These healthcare professionals not only bring good name and repute to the homeland but are also a vital source of foreign remittances which supports the national economy. Failure to acquire WFME accreditation by the deadline will have serious consequences as the medical educational programs might not be recognized internationally anymore. On the other hand, our local education and healthcare system is struggling with the issues pertaining to the quality of medical education and disparities in the induction system for postgraduate training and residency. Counting these factors together justify the need for a comprehensive educational reform in the medical education sector. However, the question is whether this new form of national licensing exam (NLE) as set by the PMC, has the capacity to make the system work more efficiently? One would argue whether it was necessary to place another exam on the face of an already overwhelmed, somewhat outdated and far from perfect examination and assessment system. Is there really a need to add redundancy into the system in the form of another exam while all other factors remain unchanged?

Currently, a high school pre-medical student has to undergo a formidable MDCAT exam to get into a medical college of either public or private sector. Once getting into a medical school, there are a numerous forms of formative and summative exams which place a tremendous burden on the shoulders of medical students. Afterwards, a graduate has yet to take another exam (FCPS-I) of the College of Physician and Surgeons Pakistan (CPSP) which is the regularizing body for postgraduate training and fellowship. Others opt for the similar type of exam (Part-I) for MD and MS programs which is taken by the individual universities. This multiple choice question (MCQ)-based theory exam is intended to determine the eligibility of a candidate to apply for a postgraduate training position. Ironically, passing candidates have yet to undergo a process for centralized induction to get into one of the public sector training programs. This cumbersome process of matching into a public sector teaching hospital with multiple barriers and redundant checkpoints is somewhat unjust, impractical and is the leading to brain-drain as more medical graduates are opting for training abroad.^4^

We find it imperative to present three critical flaws in the current policy of licensing and training-entrance level exams. Firstly, this new form of basic medical licensing exam (NLE) doesn’t complement the existing process of getting into a postgraduate residency or fellowship training. A successful candidate has yet to pass other exams (FCPS-I/MD-I/MS-I) to become eligible for a recognized fellowship training program. These exams have a structure similar to that of the NLE-I exam; none of which offer a scoring system which could count towards the process of merit formulation for the induction into residency or fellowship program. Secondly, these exams are a pass-fail in true sense with no standardized rubrics for grading that can contribute to the final merit for the centralized induction policy (CIP) for postgraduate training. Also, the CIP is heavily flawed as it has more weightage of services rendered in the rural areas’ basic health units rather than the scores achieved in any of the above mentioned exams. In fact, all passing candidates get an equal score-band from the CPSP and respective university in CIP, where basic health unit (rural) service makes the key difference overall. There is nothing much a candidate can do to improve the CIP marks if they opt not to serve in these rural areas. Thirdly, there are no clear cut guidelines as of yet, as to how this additional exit-cum-licensing exam (NLE) will be utilized to ensure and improve the quality of undergraduate medical teaching programs. In short, this current system is ill-planned and seems to be saddling the budding healthcare professionals who have a lot of potential to untap.

**Fig.1:**
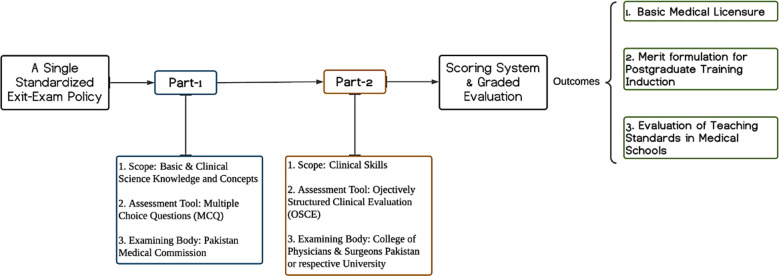
Proposed policy-model for a single, standardized licensing and residency-entrance level exam.

This calls for a simple yet robust, feasible, cost effective and user-friendly policy of basic medical licensing and postgraduate training induction. We hereby propose a more practical approach towards the restructuring of licensing and training entrance-level exam, induction policy for postgraduate training and evaluation of the standards & quality of medical education in the undergraduate teaching institutions.[Fig.1] Firstly, there should be a single-standardized exam which has a far reaching educational impact as well. This single exam should be robust enough to serve all three purposes i.e. basic medical licensure, entrance into the postgraduate training and evaluation of the teaching standards of medical colleges and universities. Like other international standardized exams, this exam can comprise two distinct parts.^5^ Part-I should aim to assess the conceptual knowledge of basic and clinical sciences through widely accepted single best answer or multiple choice question (MCQ) tool and can be administered by the highest accreditation and regulation body, i.e. the PMC. Part-II should assess application of the clinical knowledge and skills through MCQ and objectively structured clinical evaluation (OSCE) tool. This clinically oriented Part-II exam can be administered by the CPSP which can replace the FCPS-I exam, and by other universities to replace their Part-I exams for MD and MS programs. This would equally distribute the workload among the institutions and will lead to a thorough evaluation of the fresh medical graduates through different perspectives. This examination system would have more acceptability at the international level as well.^6^

Secondly, this single-standardized exam should be graded with a comprehensive scoring system so that the overall performance in these exams can contribute more towards the merit formulation for the centralized induction into fellowship training. This will foster a strong academic culture among the medical students and graduates who will invest more time in learning the concepts of basic, clinical and applied medical sciences right from the inception of their undergraduate studies. To further ensure the standard of this exam, the examining bodies can formulate a credible practice question bank for this exam which will cover all essential concepts an independent medical practitioner must know.

Lastly, we propose that this exam should lead to accountability of the quality and standard of medical education in teaching institutions across the country. Annual rankings of medical teaching institutions based on the passing percentages and scores of their candidates in the NLE can serve an effective indicator of the quality and standard of undergraduate medical education. A critical passing percentage should also be set which can serve as a warning for de-recognition of underperforming institutes barring them from inducting students in their undergraduate programs. This shall motivate the stakeholders to invest more on the educational and academic standards of their institutions while maintaining the standards set out by the WFME.

In essence, our proposed policy-model is in line with the other exit exams which are in place and successfully running worldwide. We believe this model has the potential to improve the quality of medical education in Pakistan which will be recognized worldwide. However, this can only be achieved through institutional collaboration and consensus.
